# Tau vaccines differentially target tau pathologies in Alzheimer’s disease and primary tauopathies

**DOI:** 10.1002/alz.095353

**Published:** 2025-01-09

**Authors:** Jonathan P Hulse, Madelin Whelpley, Nicole M Maphis, Julianne Peabody, Bryce Chackerian, Kiran Bhaskar

**Affiliations:** ^1^ University of New Mexico, Albuquerque, NM USA

## Abstract

**Background:**

Hyperphosphorylation and aggregation of neuronal tau protein is a primary pathological hallmark of Alzheimer’s disease (AD) and primary tauopathies. The accumulation of aggregated tau as neurofibrillary tangles (NFTs) is closely correlated with neurodegeneration and cognitive decline. Key phosphorylation sites on tau have been established as early biomarkers for disease detection and prediction, with various phosphorylation sites differentially appearing across diseases and disease stages. These phosphorylation sites may serve as valuable therapeutic targets for immunotherapeutic development with but with variable efficacy.

**Method:**

Human hippocampal brain tissue sections from Alzheimer’s disease and several primary tauopathies were immunostained using either commercial antibodies targeting specific phosphorylation sites on tau, or with immune sera obtained from mice vaccinated using a Qβ virus‐like particle (VLP) platform targeting identical tau phosphorylation sites. Immune sera were from twice vaccinated rTg4510, P301S, or C57Bl/6j mice undergoing therapeutic vaccine trials 6‐8 weeks after their first vaccination, with booster vaccinations administered two weeks apart. The vaccines used targeted the T181, S199/S202, T217, and S396/S404 tau phosphorylation sites. Immune sera were pooled from n = 5 animals each and diluted 1:500 for staining human hippocampal sections. AT8, PHF1, AT270, and Thr217 antibodies were used to compare tau histopathology. Sections were immunostained and tau visualization was performed using diaminobenzidine (DAB).

**Result:**

Immune sera from animals vaccinated with each tau vaccine successfully detected tau pathology across disease tissues similarly to commercially available antibodies. However, the range of histopathological features detected by each vaccine differed from each other within disease tissues and across diseases. Differences included detection of unique tau pathological features like neuropil threads, neuritic plaques, immature and mature NFTs, Pick bodies, and tau‐positive glial cells.

**Conclusion:**

Tau vaccines targeting various tau phosphorylation epitopes show differential recognition of tau pathology within and across brain tissue sections from Alzheimer’s disease and primary tauopathies.

